# Single-cell transcriptomics reveals stage- and side-specificity of gene modules in colorectal cancer

**DOI:** 10.21203/rs.3.rs-4402565/v1

**Published:** 2024-05-24

**Authors:** Sara Rahiminejad, Kavitha Mukund, Mano Ram Maurya, Shankar Subramaniam

**Affiliations:** University of California, San Diego; University of California, San Diego; University of California, San Diego; University of California, San Diego

**Keywords:** early tumor stage, late tumor stage, right colon, left colon, scRNA-seq, modularity, functional enrichment, WGCNA

## Abstract

**BACKGROUND:**

An understanding of mechanisms underlying colorectal cancer (CRC) development and progression is yet to be fully elucidated. This study aims to employ network theoretic approaches to analyse single cell transcriptomic data from CRC to better characterize its progression and sided-ness.

**METHODS:**

We utilized a recently published single-cell RNA sequencing data (GEO-GSE178341) and parsed the cell X gene data by stage and side (right and left colon). Using Weighted Gene Co-expression Network Analysis (WGCNA), we identified gene modules with varying preservation levels (weak or strong) of network topology between early (pT1) and late stages (pT234), and between right and left colons. Spearman’s rank correlation (*ρ*) was used to assess the similarity or dissimilarity in gene connectivity.

**RESULTS:**

Equalizing cell counts across different stages, we detected 13 modules for the early stage, two of which were non-preserved in late stages. Both non-preserved modules displayed distinct gene connectivity patterns between the early and late stages, characterized by low *ρ* values. One module predominately dealt with myeloid cells, with genes mostly enriched for cytokine-cytokine receptor interaction potentiallystimulating myeloid cells to participate in angiogenesis. The second module, representing a subset of epithelial cells, was mainly enriched for carbohydrate digestion and absorption, influencing the gut microenvironment through the breakdown of carbohydrates. In the comparison of left vs. right colons, two of 12 modules identified in the right colon were non-preserved in the left colon. One captured a small fraction of epithelial cells and was enriched for transcriptional misregulation in cancer, potentially impacting communication between epithelial cells and the tumor microenvironment. The other predominantly contained B cells with a crucial role in maintaining human gastrointestinal health and was enriched for B-cell receptor signalling pathway.

**CONCLUSIONS:**

We identified modules with topological and functional differences specific to cell types between the early and late stages, and between the right and left colons. This study enhances the understanding of roles played by different cell types at different stages and sides, providing valuable insights for future studies focused on the diagnosis and treatment of CRC.

## Background

Despite substantial progress in cancer diagnoses and treatment, it remains one of the leading causes of death worldwide. Colorectal cancer (CRC), encompassing both colon and rectal cancers, ranks as the second most common cause of cancer-related death in the US. According to the most recent estimates, approximately 153,000 individuals are diagnosed with CRC annually, leading to around 52,000 fatalities [1]. CRC is a multifactorial disease and its severity is affected by genetic, environmental, and lifestyle risk factors. While there are hereditary and non-hereditary types of CRC, the majority are non-hereditary, resulting from somatic mutations in response to environmental factors [2].

The American Joint Committee on Cancer (AJCC)’s Tumor-Node-Metastasis (pTNM) staging serves as the most robust classification schema for cancer and a foundation for post-operative clinical decision-making. This classification summarizes the extent of local invasion (pT), regional node status (pN), and the presence/absence of distant metastasis (pM) [3]. The tumor staging (pT) of CRC includes four main categories ranging from pT1 to pT4, with higher numbers indicating increasing severity. Based on the tumor’s location, CRCs are divided into the right-sided or proximal tumors (including the cecum, ascending colon, hepatic flexure, and three-quarters of the transverse colon) and the left-sided or distal tumors (including the splenic flexure, descending colon, sigmoid colon, rectosigmoid, rectum, and the remaining quarter of the transverse colon). Left-sided CRC accounts for two-thirds of all CRC cases, is often diagnosed earlier, and is more frequent in men and younger patients. In contrast, right-sided CRC is more common in women and older patients [4, 5]. Notably, tumor sided-ness has been identified as a factor influencing clinical outcomes. Consequently, ongoing studies aim to explore more effective treatments for CRC based on the site of detection [6].

In recent years, scRNA-seq has significantly contributed to quantifying the state of cancer tissue at a single-cell resolution. In CRC, single-cell resolution of the genome, transcriptome, and epigenome has elucidated the diversity within and between tumors. Dai et al. utilized scRNA-seq analysis to profile cells from cancer tissue of a CRC patient (at stage III, right colon) to provide insights into the heterogeneity of cell populations [7]. Willems et al. used scRNA-seq data to improve CRC survival prediction [8]. In [9], the transcriptional profiling of 371,223 cells from CRC tumors and adjacent normal tissues identified hubs of interacting malignant and immune cells, unveiling the logic behind the spatially organized immune-malignant cell network. However, the study lacks modular analysis across stages and sides, which will be the focus of our analysis.

**Figure 1:**
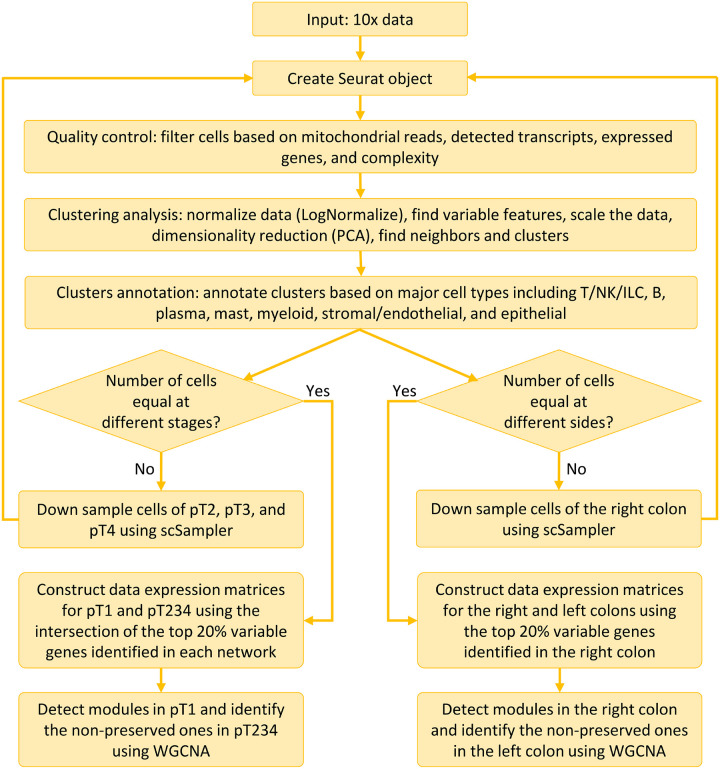
Workflow for the approach used in our analysis

In this study, we investigated events occurring in CRC, at late stages (stages pT2, pT3, and pT4 combined as pT234) compared to the early stage (pT1) and in left-sided compared to right-sided, using published scRNA-seq data containing 370,115 cells from 62 tumor and adjacent normal tissues [9]. Due to an unequal number of cells at different stages (and different sides), we down-sampled and equalized cell counts in each stage independently using *scSampler* [10]. Subsequently, all cell-level data were processed using Seurat [11–14]. We then identified gene modules with weak and strong preservation of network topology between early and late stages, and between right- and left-sided CRC, utilizing Weighted Gene Co-expression Network Analysis (WGCNA). Throughout the rest of this manuscript, we use the terms right-sided (left-sided) CRC and right (left) colon cancer interchangeably. Functional insights were derived from these less preserved modules to expand our understanding of stage- and side-specific differences in CRC [15, 16]. Figure 1 summarizes the analysis pipeline adopted in this study.

## Materials and Methods

### Single-cell RNA sequencing data acquisition and processing

Publicly available scRNA-seq data from a study on the single-cell atlas of mismatch repair-deficient (MMRd) and mismatch repair-proficient (MMRp) colorectal cancer were downloaded from GEO (accession code GSE178341). The data included 370,115 cells from 62 patients at different pathological stages of cancer (pT1 for early invasive cancer through pT4 for the growth of the tumor through the outer layer of the bowel wall) and 36 adjacent normal tissues. Of the total number of cancerous cells, there were 62,654 cells from the left colon and 194,597 cells from the right colon. We utilized the R package Seurat for processing the data [11–14].

Initially, the data were filtered at the cell level by excluding cells based on the following criteria: more than 15% mitochondrial reads, fewer than 300 detected transcripts, fewer than 500 expressed genes, and a complexity (log10 genes/UMI) of less than 70%. Gene-level filtering was also performed by excluding genes expressed in fewer than 10 cells. The data were then normalized utilizing the *LogNormalize* method and a scale factor of 10,000. The top 20% variable genes were identified, and the data were scaled. Principal Component Analysis (PCA) was performed for dimensionality reduction, with npcs = 40. The optimal dimension for clustering was selected using the following approach:

1. Determine the percentage of variance (PV) and the cumulative percentage (CP) captured by each PC.
2. Identify PCs with CP > 90 and PV < 5.
3. Calculate the difference between each PC and the subsequent PC, with a percentage change < 0.1.
4. Select the minimum value from steps 2 and 3 as the optimal number.

Uniform Manifold Approximation and Projection (UMAP) analysis was performed on the optimal number of dimensions, and cells were clustered using the Louvain algorithm (*FindClusters* function in *Seurat*) with a resolution of 2.

### Down-sampling of cells using scSampler

Due to differences in the number of cells across different stages and sides, down-sampling was performed twice. In the first part of the analysis (comparison of late vs. early stages), tumor stage 1 (pT1) with the lowest number of cells was selected as the base, and cells from other stages were down-sampled using *scSampler* [10], a Python implementation, to match the cell counts in pT1. Initially, the clustered cells (identified in the previous step) were grouped into seven major cell types: T/natural killer (NK)/innate lymphoid cell (ILC), B, plasma, mast, myeloid, stroma/endothelial, and epithelial cells, based on specific markers for each type. Then, the major cell types of each tumor stage (pT2, pT3, pT4, and normal) were down-sampled, ensuring that the sum of all cells in each stage was equal to the number of cells in pT1, and maintaining the (ratio) relative count of cells from different cell types for each stage as before down-sampling. Similarly, in the second part (comparison of the left vs. right colons), the left colon was selected as the base, and cells from the right colon were down-sampled to match the cell counts in the left colon. Following down-sampling, a subset of the original data with the down-sampled cells and all genes was selected, and the previous steps (data scaling and dimensionality reduction) were applied.

### Module detection and preservation

We utilized the R package WGCNA to identify gene modules. In the first comparison, we designated the early stage (pT1) as the reference network and late stages (pT234) as the test network. To identify the most variable genes between the two networks, we independently selected the top 20% variable genes for the early and late stages separately and used their intersection to create a list of variable genes. Subsequently, we constructed a ‘cell x gene’ expression matrix for each network and identified gene modules for the reference network using the *blockwiseModules* function of WGCNA. Preservation statistics for the detected modules were then calculated with respect to late stages. Non-preserved modules were selected based on two criteria: median rank ≥ 10 and module size ≤ 100.

In the second comparison, we designated the right colon as the reference network and the left colon as the test network. The subsequent steps mirrored those of the first comparison, with the exception that, the list of variable genes consisted of the top 20% variable genes from the reference network.

### Changes in non-preserved modules between reference and test networks

Non-preserved modules were selected based on the median rank plot, as described in the original publication [17]. To visualize these modules, the following steps were carried out separately for the reference and test networks.

1. Construct a gene by cell expression matrix.
2. Select a subset of cells for which the average expression of each cell across all genes of module *x* is greater than the average expression of that cell across all genes of all other modules (excluding modules *x* and grey).
3. Extract a sub-matrix using the resulting cells and the genes of module *x*.
4. Calculate the correlation network adjacency from the sub-matrix and raise it to the power beta (chosen for the network to exhibit a scale-free topology).

The resulting network was then visualized in a circle plot, where edge weight represented the co-expression value between two genes, and node (gene) connectivity (or weighted-degree) indicating the sum of edge weights connected to that node. Nodes were colored based on the z-score between reference and test networks, transitioning from blue (−2 or lower) to white (0) and to red (2 or greater). Functional analysis on the modules or a subset of the nodes (genes) was conducted using Enrichr [18–20].

### Spearman’s rank correlation

To assess the similarity/dissimilarity of non-preserved modules between reference and test networks in terms of node connectivity, we employed Spearman’s rank correlation coefficient (*ρ*). Initially, we ranked the genes of the non-preserved modules in reference and test networks based on their connectivity. We then utilized the following equation to calculate the Spearman’s rank correlation coefficient, which ranges between − 1 and 1. A lower (larger) value indicates less (more) similarity between the genes’ connectivity in the two networks.

1 $$\rho =1-\frac{6\sum d_{i}^{2}}{n(n^{2}-1)}$$

where *d*_*i*_ represents the difference between the ranks of node *i* in the two networks, and *n* denotes the total number of genes in that module.

### Normalized two sample z-test for genes

z-score for a gene between two data sets was calculated using a two-sample z-test.

2 $$z=\frac{\left({\bar{x}}_{1}-{\bar{x}}_{2})-(\mu_{1}-\mu_{2}\right)}{\sqrt{\frac{\sigma_{1}^{2}}{n_{1}}+\frac{\sigma_{2}^{2}}{n_{2}}}}$$

in which ${\bar{x}}_{1}$ and ${\bar{x}}_{2}$ represent the sample mean of the first and second samples, respectively,*μ*_1_ and *μ*_2_ represent the mean of the first and second populations, $\sigma_{1}^{2}$ and $\sigma_{2}^{2}$ are the population variances in the first and second populations, and *n*_1_ and *n*_2_ represent the sample sizes of the first and second groups, respectively. Assuming the null hypothesis *μ*_1_ = *μ*_2_, the z-score is calculated using the following equation:

3 $$z=\frac{{\bar{x}}_{1}-{\bar{x}}_{2}}{\sqrt{\frac{\sigma_{1}^{2}}{n_{1}}+\frac{\sigma_{2}^{2}}{n_{2}}}}$$

## Results and Discussion

### Down-sampling to equalize the number of cells at different stages and sides

**Figure 2:**
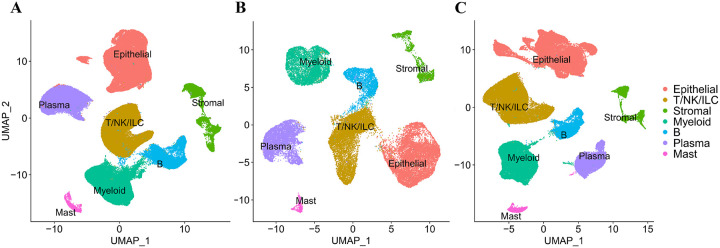
UMAP plot for all cells. **A** Before down-sampling. **B** After down-sampling late stage (pT234) cells. **C** After down-sampling right colon cells

The CRC dataset utilized in this study contained varying numbers of cells across different stages and sides. The down-sampling process was performed separately on the major cell types for the late stages to align the cell counts with those of the early stage, and on the major cell types for the right colon to align the cell counts with those of the left colon (see Materials and Methods). Cell counts in each major cell type before and after down-sampling at different stages and sides are listed in Supplementary Tables S1 and S2. Feature plots and dot plots for cell markers are shown in Supplementary Figures S1 and S2. UMAP plots, depicting cells both before and after down-sampling, clearly highlight the separation between major cell types (Figs. 2A–C).

### Comparison of late vs. early stages

**Figure 3:**
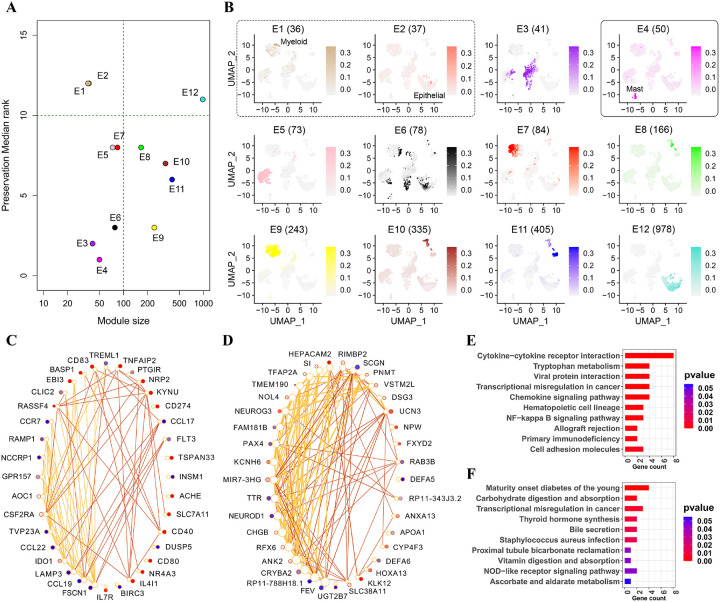
Topological and functional analysis of the modules detected for the early stage (pT1). **A** Median rank of module preservation between the early (reference) and late stages (test). **B** Cellcoverage of various modules detected for the early stage. The modules are arranged in ascending order based on their sizes, from the smallest to the largest. The number after each module’s name represents the number of genes in that module. Non-preserved (or preserved) modules are outlined by a dashed (or solid) black rectangle. C-D Circle plot for top 10% unique edges of modules E1 and E2, respectively. Each yellow (or brown) edge represents the co-expression value between the two corresponding genes in the early stage (or late stages). The white (colored) circle near each gene represents the sum of weights of edges connected to that gene in the early (or late) stages. Circles are colored based on the z-score between late stages vs. the early stage, ranging from blue for z-score ≤ −2, white for z-score = 0, to red for z-score ≥ 2. E-F Top 10 enriched KEGG pathways (based on p-values) for modules E1 and E2, respectively.

After down-sampling, the early stage (pT1) comprised 7,540 cells and the late stages (pT234) had 22,620 (7,540*3) cells. The intersection of the top 20% variable genes from the two stages resulted in 3,107 unique genes. Constructing a data expression matrix with these genes and cells from the early stage, we identified 13 modules for the early stage (labeled E1 through E13). Among these modules, E1 and E2 exhibited lower preservation, while E4 demonstrated the highest preservation in late stages, as determined by median rank statistics (Fig. 3A). Cell coverages of all modules in UMAP space are also shown in Fig. 3B. Comparing these plots with Fig. 2B indicated that module E1 captured myeloid cells, module E2 captured epithelial cells, and module E4 captured mast cells. In the following section, we analyze non-preserved and preserved modules from a functional perspective.

### Non-preserved modules are functionally distinct between the early and late stages

As mentioned in the previous section, modules E1 and E2 were not preserved in the late stages. Spearman’s rank correlation which assigns values between 0 (least similar) and 1 (most similar), also confirmed the lack of preservation in gene connectivity for these modules between the early and late stages (0.63 for E1 and 0.42 for E2). Figures 3C and 3D display the top 10% of unique edges in the early and late stages for modules E1 and E2 in circle plots, respectively. These figures highlight changes in gene connectivity, average edge weights, and up/down regulation of the genes (late vs. early) for both modules. Larger edge weights were noted in the early stage for both modules. The difference in gene connectivity between the early and late stages is not very high in module E1. In contrast, for module E2, the genes exhibit significantly higher connectivity in the early stage compared to late stages. Functional roles of these modules will be emphasized in the following sections.

Module E1 captured myeloid cells, as evident in Figs. 2B and 3B, constituting the most abundant cell population in the tumor microenvironment (TME) and playing a key role in CRC tumor pathogenesis. Studies have established that myeloid cells are critically involved in regulation of tumor progression and metastasis [21]. Specifically, genes of module E1 were enriched in mature dendritic cells, showing expression changes between the early and late stages. Dendritic cells (DCs), a distinct myeloid cell lineage, are pivotal in initiating the adaptive immune response and stand as a significant target for cancer immunotherapy. These genes were mostly associated with cytokine-cytokine receptor interaction, tryptophan metabolism, and chemokine signaling pathways, as illustrated in Fig. 3E. It has been demonstrated that cytokines can stimulate myeloid cells to participate in angiogenesis, the formation of new blood vessels, which is critical for tumor growth and metastasis [22]. Tryptophan metabolites, products of tryptophan metabolism, can modulate the polarization and function of tumor-associated macrophages (TAMs) [23]. Studies have shown that tryptophan metabolism may contribute to the TAMs polarization towards a pro-tumorigenic phenotype, promoting tumor progression [24]. Additionally, chemokine signaling, another crucial pathway within this module, is often associated with inflammation, a hallmark of colorectal cancer [25]. Myeloid cells respond to chemokine signals by releasing additional inflammatory mediators, contributing to the perpetuation of an inflammatory and pro-tumorigenic microenvironment [26].

Within module E1, the gene with the highest connectivity in the early stage was *FSCN1* (as shown in Fig. 3C), which has been shown to promote cancer cell migration and invasion by contributing to the formation of cellular protrusions, including filopodia and invadopodia [27]. It could also serve as a potential novel marker or therapeutic target for patients with aggressive forms of colorectal adenocarcinoma [28]. The gene with the second-highest connectivity was *CCL19*, which is abundantly expressed in T-cell zones such as lymph nodes and the thymus. It plays a crucial role in regulating immune responses by controlling the migration of DCs and T cells into secondary lymphatic tissues [29, 30]. Moreover, CCL19 functions as a factor supporting tumors by inducing inflammation, cell growth and, metastasis [31]. Lastly, *LAMP3*, the gene with the third-highest connectivity, is recognized for its involvement in tumor metastasis and drug resistance, significantly contributing to tumor cell proliferation, migration, and invasion [32].

The non-preserved module E2 is primarily associated with a small subset of epithelial cells, specifically goblet cells (GCs) characterized by genes *DEFA6, KLK12, HEPACAM2, ANXA13, NEUROG3*, and *UGT2B7*. GCs are intestinal mucosal epithelial cells serving as the primary site for nutrient digestion and mucosal absorption [33]. Their primary function involves synthesizing and secreting mucins, which have demonstrated significance in various stages of CRC’s metastatic processes [34]. The genes within this module were predominantly enriched in pathways such as carbohydrate digestion and absorption, transcriptional misregulation in cancer, and bile secretion (as shown in Fig. 3F). Carbohydrate breakdown can produce short-chain fatty acids, which are metabolites that can influence the gut microenvironment. Changes in carbohydrate metabolism may impact the interaction between epithelial cells and gut microbiota, potentially influencing CRC development and progression [35]. The transcriptional misregulation pathway, another crucial pathway, could affect communication between epithelial cells and the tumor microenvironment, influencing surrounding stromal and immune cells. This interaction is integral to the progression and behavior of colorectal tumors [36]. Within this pathway, *DEFA5* and *DEFA6* exhibited downregulation in late stages compared to the early stage. *DEFA5* is closely associated with colorectal adenocarcinoma, and its high expression correlates with a more favorable prognosis [37]. *DEFA6* has been demonstrated to support proliferation, migration, invasion, and colony formation of CRC cell lines *in vitro* [38]. The bile secretion pathway is another important pathway associated with module E2. Bile acids serve as potent stimulators of CRC initiation by damaging colonic epithelial cells. They promote CRC progression through various mechanisms, including apoptosis inhibition, enhancement of cancer cell proliferation, invasion, and angiogenesis [39].

Additional markers previously associated with crucial roles in CRC were identified within this module. For instance, *TTR* exhibited higher connectivity in the early stage compared to late stages (Fig. 3D), possessing cytokine functions and contributing to the stimulation of myeloid cell differentiation, known to play an important role in the tumor environment [40]. Similarly, *HEPACAM2*, showing the same pattern in gene connectivity, is involved in immune response progression, chemokine signaling, Ras signaling, and Hematopoietic cell lineage pathways [41]. Its elevated expression has been associated with a better prognosis and the reduced risk of death in patients with COAD, as indicated by various models. Studies suggest its potential as a diagnostic and prognostic biomarker for colon adenocarcinoma [41]. *NEUROD1*, another gene with higher connectivity in the early stage, is recognized for its high expression in CRC. Silencing *NEUROD1* results in the activation of p21, a pivotal cell cycle regulator. This activation leads to G2-M phase arrest, effectively suppressing the proliferation of colorectal cancer cells and diminishing their potential for colony formation [42].

### Deciphering the role of the preserved module in colorectal cancer

From the median rank plot (Fig. 3A), it is evident that module E4, capturing the majority of mast cells (MCs), remained highly preserved in the comparison of late vs. early stages. Spearman’s rank correlation of gene connectivity (0.84) further validated this module’s preservation in gene connectivity. MCs serve as primary sentinels in the gut and play an important role in host defense by maintaining immune system homeostasis and orchestrating local inflammation. While MCs contribute to the transition from chronic inflammation to cancer, their precise role in tumor initiation and growth remains controversial. According to recent studies [43, 44], MC-derived mediators can exert pro-tumorigenic functions, causing tumor progression by inducing angiogenesis and promoting tissue remodeling through changes in composition of the extracellular matrix. Conversely, these mediators can also exert anti-tumorigenic functions, limiting tumor growth by promoting pro-inflammatory pathways and thereby impairing the tumor progression.

The genes of module E4 were mostly enriched in the renin-angiotensin system (RAS), FcεRI signaling, and histidine metabolism pathways. Histamine, released by mast cells as a mediator, may modulate the chronic inflammatory response associated with developing neoplasm [45]. Notably, studies have demonstrated an inverse relationship between circulating histidine levels and the risk of colorectal cancer [46]. In the histidine metabolism pathway, *MAOB* and *HDC* exhibited higher z-scores in late stages compared to the early stage. Additionally, two significant genes enriched in the FcεRI signaling pathway, *IL5* and *IL13*, are noteworthy. Studies have indicated that *IL5* possesses antitumor properties in CRC and tends to be elevated in mid CRC stages (pT2 and pT3) [47]. Notably, we observed a higher z-score for *IL5* in late stages compared to the early stage. Similarly, IL13 was upregulated in late stages (higher z-score). It has been shown that *IL13* signaling may play a role early in intestinal stem cell self-renewal and homeostasis, potentially contributing to a tumorigenic microenvironment [48, 49].

### Comparison of left vs. right colons

**Figure 4:**
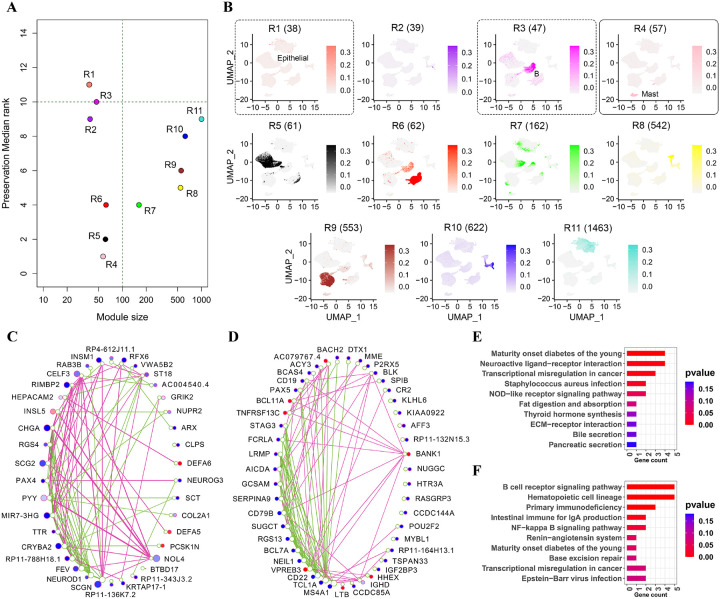
Topological and functional analysis of the modules detected for the right colon. **A** Median rank of module preservation between the right (reference) and left colon (test). **B** Cell coverage of different modules detected for the right colon. The modules are arranged in ascending order based on their sizes, from the smallest to the largest. The number after each module’s name represents the number of genes in that module. Non-preserved (or preserved) modules are highlighted by a dashed (or solid) blue rectangle around them. **C-D** Circle plot for top 10% unique edges of modules R1 and R3, respectively. Each green (or crimson red) edge represents the co-expression value between the two corresponding genes in the right colon (or left colon). The left (or right) circle near each gene represents the sum of weights of edges connected to that gene in the right (or left) colon. Circles are colored based on the z-score between the left and right colon, ranging from blue for z-score ≤ −2, white for z-score = 0, to red for z-score ≥ 2. **E-F** Top 10 enriched KEGG pathways (based on p-values) for modules R1 and R3, respectively.

After down-sampling (see Materials and Methods), both the right and left colons contained 46,379 cells. Using the top 20% variable genes from the right colon (5,150 genes), we constructed a data expression matrix and identified 12 modules. Among these, modules R1 and R3 showed less preservation, while module R4 exhibited the highest preservation in the left colon, as determined by median rank statistics (Fig. 4A). Cell coverages of all modules in UMAP space are also shown in Fig. 4b. Comparing these plots with Fig. 2C indicated that module R1 captured a small fraction of epithelial cells, myeloid cells, module R3 captured B cells, and module R4 captured mast cells. In the following section, we analyze non-preserved and preserved modules from both a topological and functional standpoint.

### Non-preserved modules are functionally distinct between the right and left colons

As mentioned in the previous section, modules R1 and R3 were not preserved in the left colon. Spearman’s rank correlation also confirmed a lack of preservation in gene connectivity for these modules between the right and left colons to a certain extent (0.48 for R1 and 0.73 for R3). Figures 4C and 4D display the top 10% of unique edges in the right and left colons for modules R1 and R3 in circle plots, respectively. These figures illustrate changes in gene connectivity, average edge weights, and up/down regulation of the genes between the right and left colons for both modules. Larger edge weights were observed in the left colon for R1 and in the right colon for R3. Moreover, a majority of the genes in both modules were predominantly downregulated in the left colon compared to the right colon, as reflected in negative z-scores. The functional roles of these modules will be emphasized in the following sections.

Module R1 captured a small fraction of epithelial cells (similar to module E2 in late vs. early comparison) and was mostly enriched for neuroactive ligand-receptor interaction, transcriptional misregulation in cancer, and NOD-like receptor signaling pathways based on KEGG (Fig. 4E). Neuroactive ligand-receptor interactions can affect the proliferation and survival of epithelial cells. Previous studies have revealed a significant association between this pathway and the development of colorectal cancer, as indicated by GO and KEGG enrichment analysis [50]. The role of transcriptional misregulation in cancer has been previously discussed in the comparison of late vs. early stages (module E1). NOD-like receptor signaling, another enriched pathway for module R1, has been shown to be involved in inflammation-associated tumorigenesis, angiogenesis, cancer cell stemness, and also chemoresistance [51].

Some genes within module R1, such as *CHGA, CELF3, RIMBP2, SCG2, TTR, DEFA5*, and *DEFA6*, play significant roles in CRC. Notably, *CHGA, CELF3, RIMBP2, SCG2*, and *TTR* displayed significant differences in gene connectivity between the right and left colons compared to other genes (Fig. 4C), all exhibiting downregulation in the left colon relative to the right colon. *CHGA* has been implicated in the initiation of colon cancer, and according to our analysis, it could serve as a potential biomarker for detecting left-sided colon cancer [52]. Another noteworthy gene, *CELF3*, has shown distinct expression patterns in patients with mCRC [53]. *SCG2*, with higher gene connectivity in the left colon, is identified as an independent prognostic predictor in CRC. *SCG2*’s high expression correlated with poor survival and advanced clinical stage (or subsequently right-sided colon) in CRC patients [54]. Similarly, *TTR* has been shown to have significantly lower expression in patients with mCRC compared to patients without mCRC, making it a potential indicator to evaluate the occurrence and prognosis of CRC [55]. *DEFA5* and *DEFA6*, two notable genes, were upregulated in the left colon compared to the right colon, along with a few other genes. Arijs et.al observed a marked upregulation of *DEFA5* and *DEFA6* expression in the inflamed colon of patients with ulcerative colitis [56].

Module R3 captured the majority of B cells (Fig. 4B). B cells have a crucial role in maintaining human gastrointestinal health through the production of IgA and IgM, contributing to the protection of the epithelial barrier [57]. Tumoral B cells are effective in antigen presentation and co-stimulation, activating tumoricidal T cells and influencing cancer cell death. However, it has been observed that a high density of tumoral B cells predicts a favorable clinical outcome only in patients with right-sided colon cancer, as opposed to those with left-sided colon cancer [58]. The genes within module R3 were mostly enriched in B cell receptor signaling, hematopoietic cell lineage, and primary immunodeficiency pathways based on KEGG (Fig. 4F). Hematopoietic cell lineage is a biological process involving the formation and development of blood cells, including B cells, from hematopoietic stem cells. Hematologic cells play a central role in tumor growth and progression in the solid tumor microenvironment, where cancer stem cells can differentiate into hematopoietic cells [59]. The primary immunodeficiency pathway in cancer involves immune surveillance, in which B cells play a crucial role. Studies have shown that defects in this pathway may compromise the effectiveness of immune surveillance, potentially allowing cancer cells to evade detection [60].

A majority of the genes within module R3 exhibited higher connectivity in the right colon compared to the left colon and were mostly downregulated in the left colon relative to the right (Fig. 4D). *MS4A1*, the gene with the highest gene connectivity in the right colon, has been shown to be downregulated in colorectal carcinoma and its expression is positively correlated with CRC patient survival [61]. *CD19*, *CD22*, and *CD79B*, which were downregulated in the left colon compared to the right and enriched in the B cell receptor signaling pathway, play a central role in the initiation and development of colon cancer [62]. Another gene exhibiting differences in gene connectivity between the right and left colons is *TCL1A*. Studies have shown higher expression of *TCL1A* in CRC cancerous stages compared to normal, correlating with tumor differentiation and clinical stage [63]. *TCL1A* serves as a useful biomarker for the prognostic evaluation of patients at stages 2 and 3 of CRC [64]. *RGS13*, another notable gene, has been shown in studies to regulate mast and T cell migration and activation [65], suggesting that RGS proteins may also serve as a prognostic factor in CRC diagnosis [66].

### Preserved module is enriched for immune associated signaling pathways

The median rank plot (Fig. 4A) indicates that module R4 was highly preserved in the left vs. right colon comparison. The validation of gene connectivity preservation within this module was further supported by a Spearman’s rank correlation of 0.97. Similarly, akin to the module preserved in the late vs. early stages comparison (module E4), module R4 also encompassed mast cells, known for their controversial role in CRC [43]. The functional enrichment of the genes in R4 is quite similar to that of E4, with the genes mostly enriched for the RAS, FcεRI signaling, and JAK/STAT signaling pathways. Studies have demonstrated that most immune cells, including mast cells, express components of the RAS. As a result, these components emerge as potential targets for modulating tumor-in filtrating immune cells, presenting a mechanism of tumor control by the renin–angiotensin system inhibitors (RASi) [67]. RAS also plays a pivotal role in CRC metastasis [68]. FcεRI is a receptor primarily found on the surface of mast cells and basophils, and it is involved in recognizing and binding to the Fc region of immunoglobulin E (IgE) antibodies. Upon activation, the FcεRI signaling pathway triggers a cascade of intracellular events, leading to the release of various inflammatory mediators and cytokines [69]. It has been shown that the JAK/STAT signal transduction pathway can rapidly transmit extracellular signals to the nucleus, playing a crucial role in activating oncogenes and deactivating tumor suppressor genes in the context of colon cancer [70].

## Conclusion

Our study aimed to investigate cell-level differences between early and late stages, and between right and left sides of CRC, using scRNA-seq data. We employed WGCNA to identify gene modules associated with CRC progression and sided-ness, and conducted preservation analysis to detect modules with varying preservation levels in network topology. Functional analysis enriched the study by uncovering biological processes potentially linked to different stages and sides. In the late vs. early stages comparison, we identified thirteen modules in the early stage, two of which were non-preserved in late stages. One of the non-preserved modules captured myeloid cells, crucial in the tumor microenvironment potentially impacting cancer immunotherapy. The other was linked to epithelial cells, specifically goblet cells, emphasizing its relevance to mucosal functions and nutrient absorption. In the left vs. right comparison, we identified twelve modules in the right colon, of which, two were not preserved in the left colon. One was associated with epithelial cells, while the other was predominantly linked to B cells, indicating variation in cell composition between the two sides and the complex interplay within the tumor microenvironment. In both comparisons, the preserved module largely captured mast cells. Notably, gene networks in mast cells were largely conserved, regardless of stage or side, underscoring the role and importance of mast cells in CRC and reshaping the tumor microenvironment. These findings contribute to our understanding of the CRC biology and provide new avenues for future studies to enhance diagnostic approaches, uncover novel therapeutic targets, and improve patient outcomes.

## Data Availability

The dataset analyzed in the current study is available in the Gene Expression Omnibus (GEO) repository with the accession code GSE178341 [https://www.ncbi.nlm.nih.gov/geo/query/acc.cgi?acc=GSE178341].

## Abbreviations

AJCC: American Joint Committee on Cancer
CRC: Colorectal Cancer
DC: Dendritic Cell
GC: Goblet Cell
KEGG: Kyoto Encyclopedia of Genes and Genomes
MC: Mast Cell
MMRd: Mismatch Repair-deficient
MMRp: Mismatch Repair-proficient
PCA: Principal Component Analysis
RAS: Renin-Angiotensin System
scRNA-seq: single-cell RNA sequencing
TAM: Tumor-Associated Macrophage
TME: Tumor Microenvironment
UMAP: Uniform Manifold Approximation and Projection
WGNCA: Weighted Gene Co-expression Network Analysis
